# Supplementary material for: Beta-cell specific Insr deletion promotes insulin hypersecretion and improves glucose tolerance prior to global insulin resistance
Source: Nat Commun. 2022 Feb 8;13:735. doi: 10.1038/s41467-022-28039-8 (PMC8826929; doi:10.1038/s41467-022-28039-8)
Supplement: Supplementary file 1 — Supplementary Information [file 41467_2022_28039_MOESM1_ESM.pdf]

Supplementary figures

Title

Beta-cell specific *Insr* deletion promotes insulin hypersecretion and improves glucose tolerance prior to global insulin resistance

Author list

Søs Skovsø[1], Evgeniy Panzhinskiy[1], Jelena Kolic[1], Haoning Howard Cen[1], Derek A. Dionne[1], Xiao-Qing Dai[2], Rohit B. Sharma[3], Lynda Elghazi[4], Cara E. Ellis[1], Katharine Faulkner[5], Stephanie A.M. Marcil[1], Peter Overby[1], Nilou Noursadeghi[1], Daria Hutchinson[1], Xiaoke Hu[1], Hong Li[1], Honey Modi[1], Jennifer S. Wildi[1], J. Diego Botezelli[1], Hye Lim Noh[6,7], Sujin Suk[6], Brian Gablaski[7,8], Austin Bautista[2], Ryekjang Kim[2], Corentin Cras-Méneur[9], Stephane Flibotte[10], Sunita Sinha[11], Dan S. Luciani[12], Corey Nislow[11], Elizabeth J. Rideout[1], Eric N. Cytrynbaum[5], Jason K. Kim[6,8] Ernesto Bernal-Mizrachi[13], Laura C. Alonso[3], Patrick E. MacDonald[2], James D. Johnson[1]*

Affiliations

[1] Diabetes Research Group, Life Sciences Institute, and Department of Cellular and Physiological Sciences. University of British Columbia, Vancouver, BC, Canada

[2] Alberta Diabetes Institute and Department of Pharmacology, University of Alberta, Edmonton, Canada

[3] Division of Endocrinology, Diabetes and Metabolism and the Weill Center for Metabolic Health, Weill Cornell Medicine, New York, NY, USA

[4] Department of Ophthalmology and Visual Sciences, University of Michigan Kellogg Eye Center, Ann Arbor, MI, USA

[5] Department of Mathematics, University of British Columbia, Vancouver, BC, Canada

[6] Program in Molecular Medicine University of Massachusetts Medical School, Worcester, MA, USA

[7] Present address: Charles River Laboratories, Shrewsbury, MA, USA.

[8] Division of Endocrinology, Diabetes and Metabolism, Department of Medicine, University of Massachusetts Medical School, Worcester, MA, USA

[9] Department of Internal Medicine, Division of Metabolism, Endocrinology and Diabetes, University of Michigan, Ann Arbor, MI, USA

[10] UBC Life Sciences Institute Bioinformatics Facility, University of British Columbia, Vancouver, BC, Canada

[11] UBC Sequencing and Bioinformatics Consortium, Pharmaceutical Sciences, University of British Columbia, Vancouver, BC, Canada

[12] BC Children's Hospital Research Institute, Department of Surgery, Faculty of Medicine, University of British Columbia, Vancouver, BC, Canada

[13] Division of Endocrinology, Diabetes and Metabolism, University of Miami Miller School of Medicine and Miami VA Health Care System, Miami, FL, USA

*Email: James.d.johnson@ubc.ca, Twitter @JimJohnsonSci ; https://orcid.org/0000-0002-7523-9433

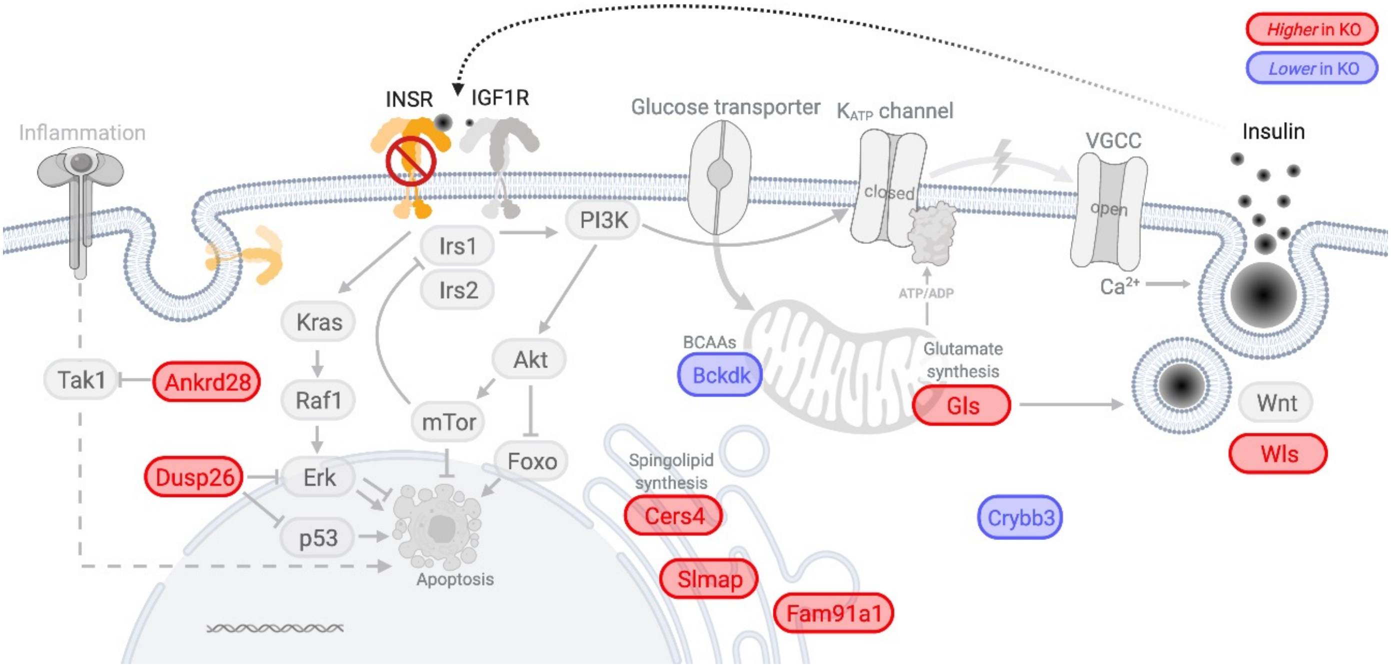

Fig. S1. Possible roles of differentially expressed genes in beta-cells lacking Insr. The diagram depicts functions of genes differentially expressed between beta-cells of wildtype controls and beta-cell specific Insr KO mice (both sexes pooled). High and low expression levels are indicated by red and blue, respectively.

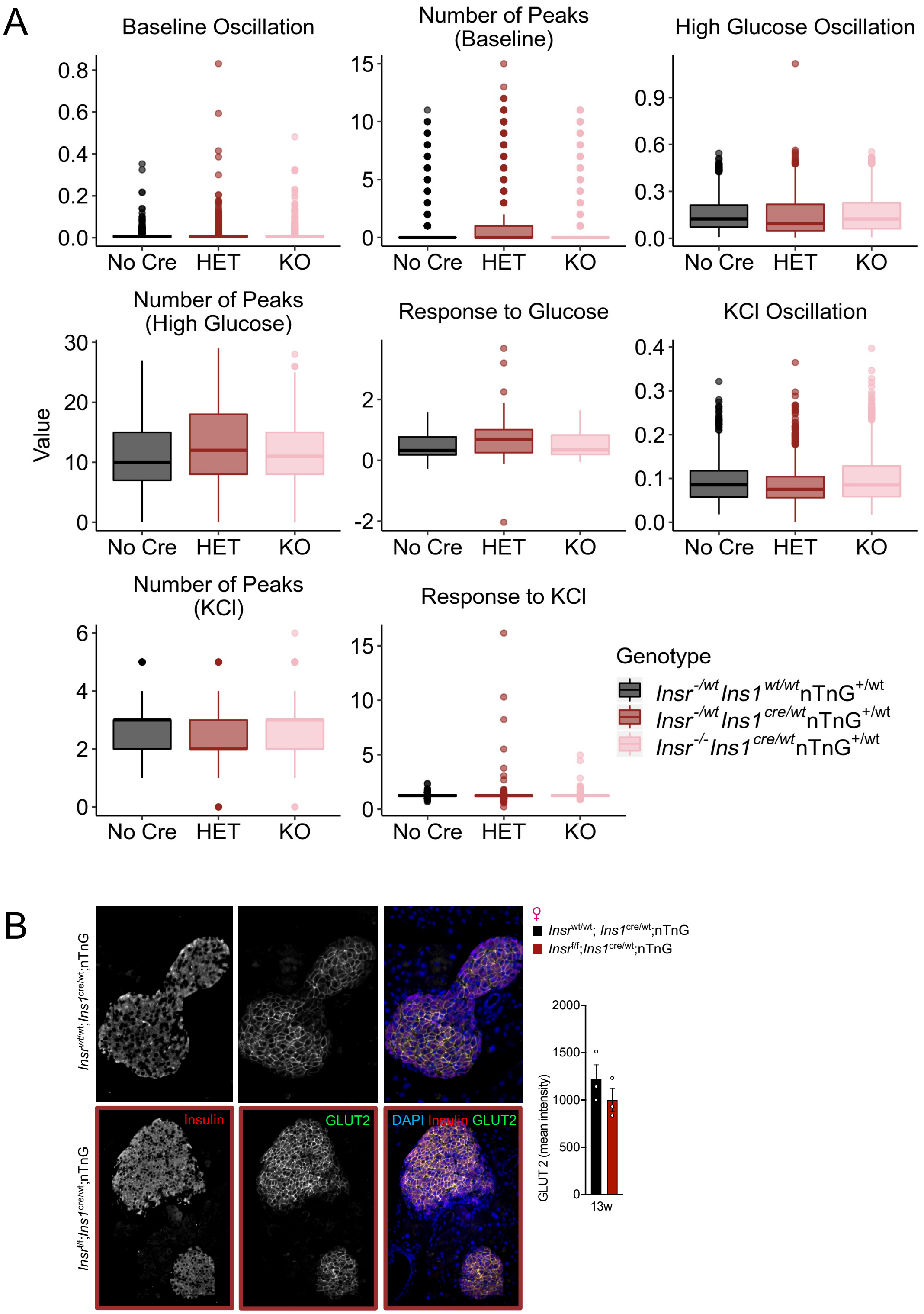

Fig. S2. Additional quantification of dynamic Ca^{2+} responses. (A) Responses to glucose or KCl were defined as the median high glucose (15mM) or KCl (30mM) signal above baseline glucose (3mM), respectively, and normalized to the maximum response to KCl above baseline. Same data as Figure 3G (n=3523 cells, from 6 mice, 2 from each group). High glucose-stimulated Ca^{2+} oscillations, Ca^{2+} oscillation in low glucose, and KCl-stimulated Ca^{2+} oscillation were defined as the median absolute deviation (MAD) during the high glucose, low glucose or KCl exposures, respectively, normalized to the maximum response to KCl above baseline. Boxplot: Minima: minimum outliers; Maxima: maximum outliers; Centre: median; Bounds of box: first to third quartile; Whiskers: the upper whisker extends from the hinge to the largest value no further than 1.5 * IQR from the hinge (where IQR is the inter-quartile range, or distance between the first and third quartiles). The lower whisker extends from the hinge to the smallest value at most 1.5 * IQR of the hinge. **(B)** Representative images and quantification of Slc2a2 (Glut2) in islets from sectioned pancreas from a LFD-fed 13 week old female control and β*Insr*^{KO} mice (Mean intensity from 5-9 islets per mouse, n=3 mice per group, sections stained side by side). Data are presented as mean values +/-SEM . Unpaired two sided student's t-test.

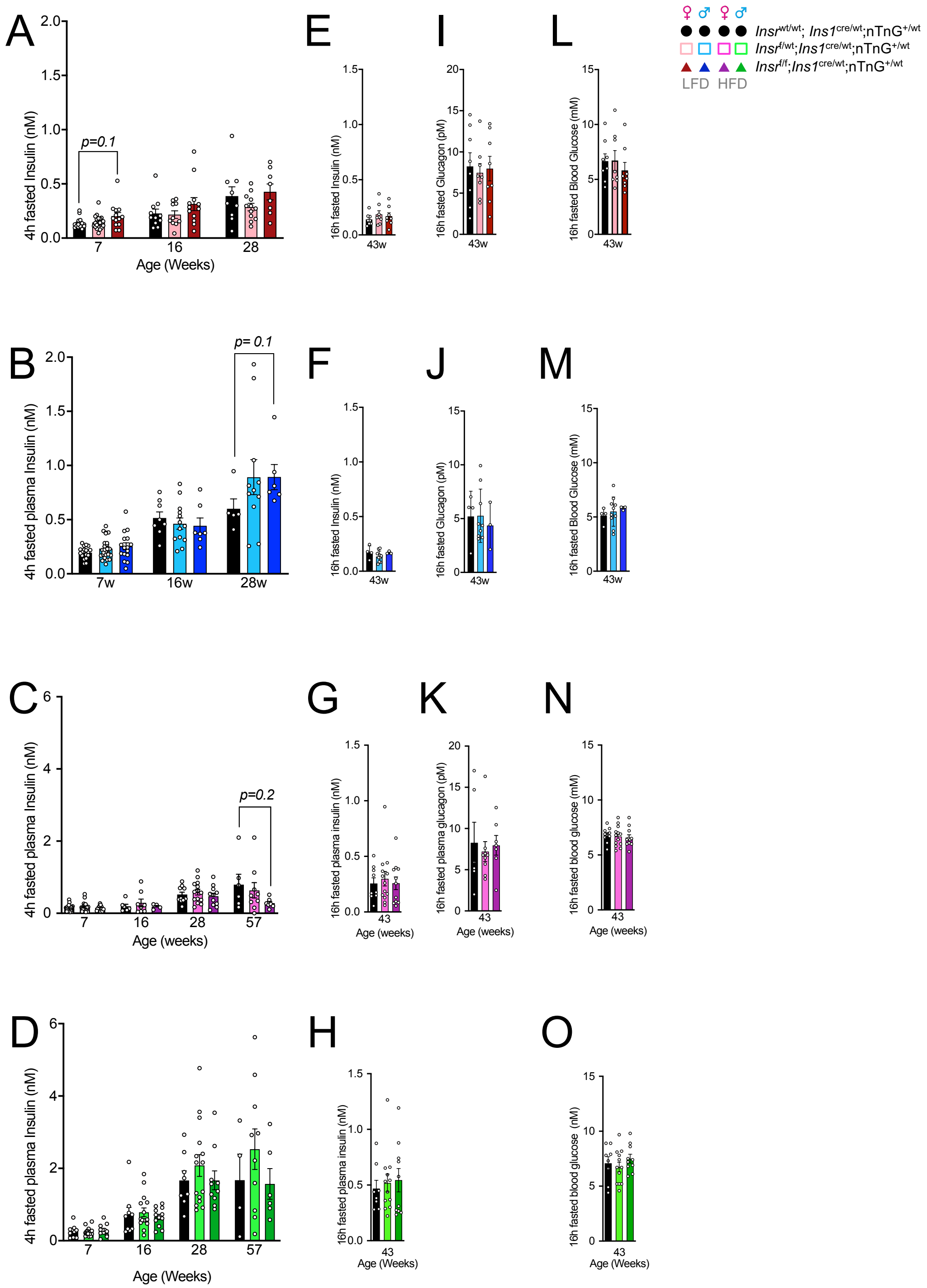

Fig. S3. Fasting insulin, glucagon, and glucose levels. (A-D) Circulating plasma insulin levels after a 4-hour fast (Data were analysed by a fitted mixed-effects model with correction for multiple comparisons using Dunnett's method), **(E-H)** or a 16-hour fast (Data were analyzed by 1-WAY ANOVA). **(I-K)** plasma glucagon levels after a 16-hour fast. Data were analyzed by 1-WAY ANOVA. **(L-O)** Blood glucose concentration after a 16-hour fast in control, β*Insr*^{HET}, and β*Insr*^{KO} mice at multiple ages. Data were analyzed by 1-WAY ANOVA. All data are presented as mean values +/-SEM (n=4-3).

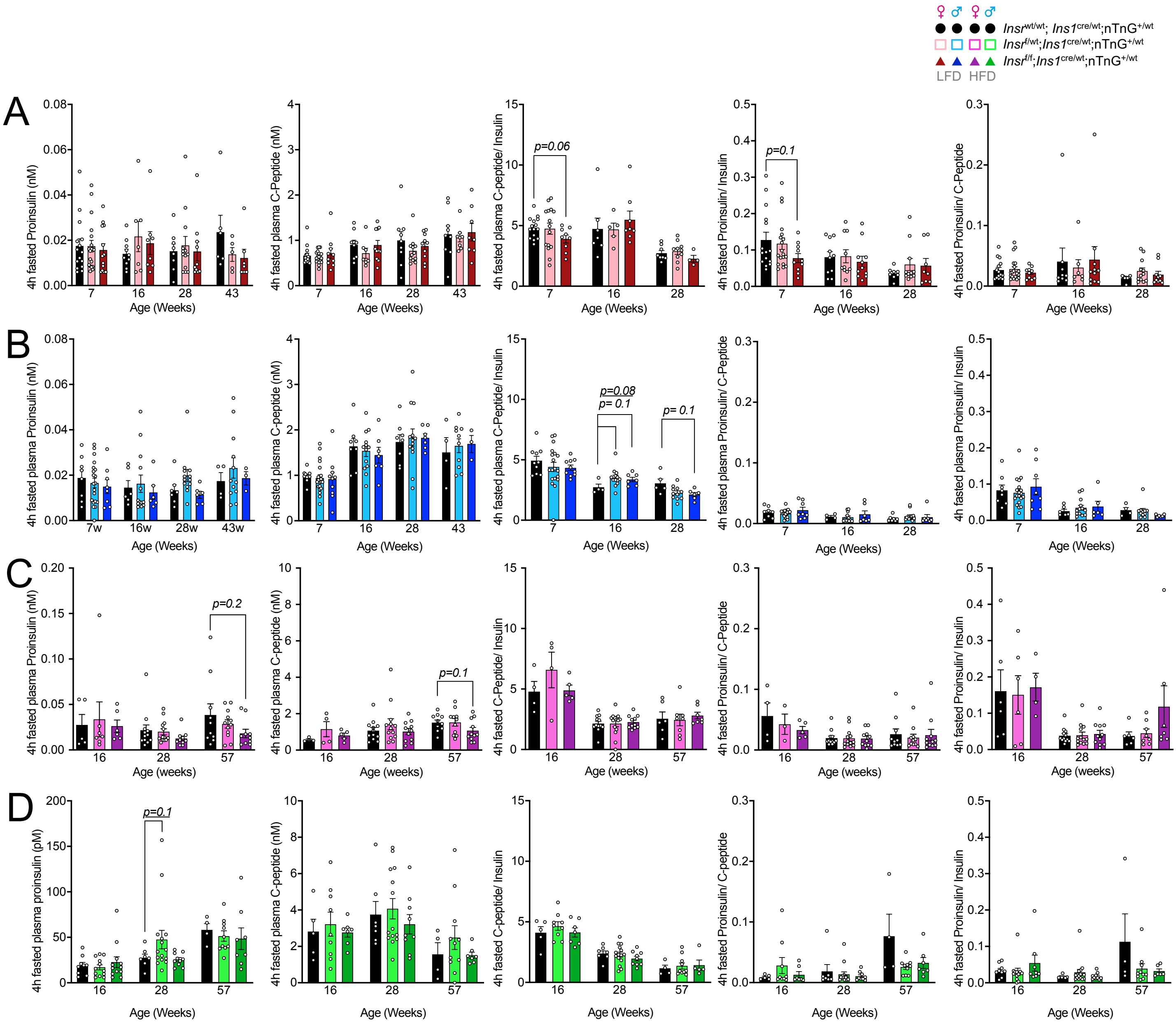

Fig. S4. Fasting proinsulin and C-peptide. (A-D) Circulating plasma proinsulin and C-peptide levels after a 4-hour fast, and their associated ratios in control, β*Insr*^HET, and β*Insr*^KO mice at multiple ages (n=3-19). Data were analysed by a fitted mixed-effects model with correction for multiple comparisons using Dunnett's method. Data are presented as mean values +/-SEM.

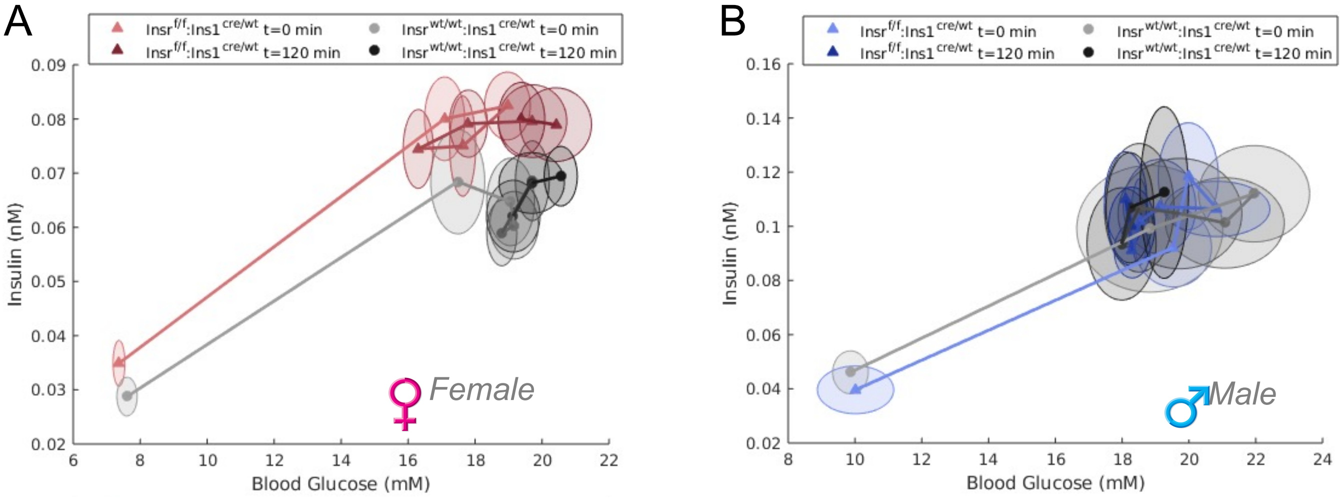

Fig. S5 Mathematical modeling of hyperglycemic clamp data. (A,B) Relationship between insulin and glucose during the hyperglycemia clamps over time in female and male mice. Data from the clamp studies were used to define a beta-cell insulin sensitivity term that was included in a modified Topp model (see main text).

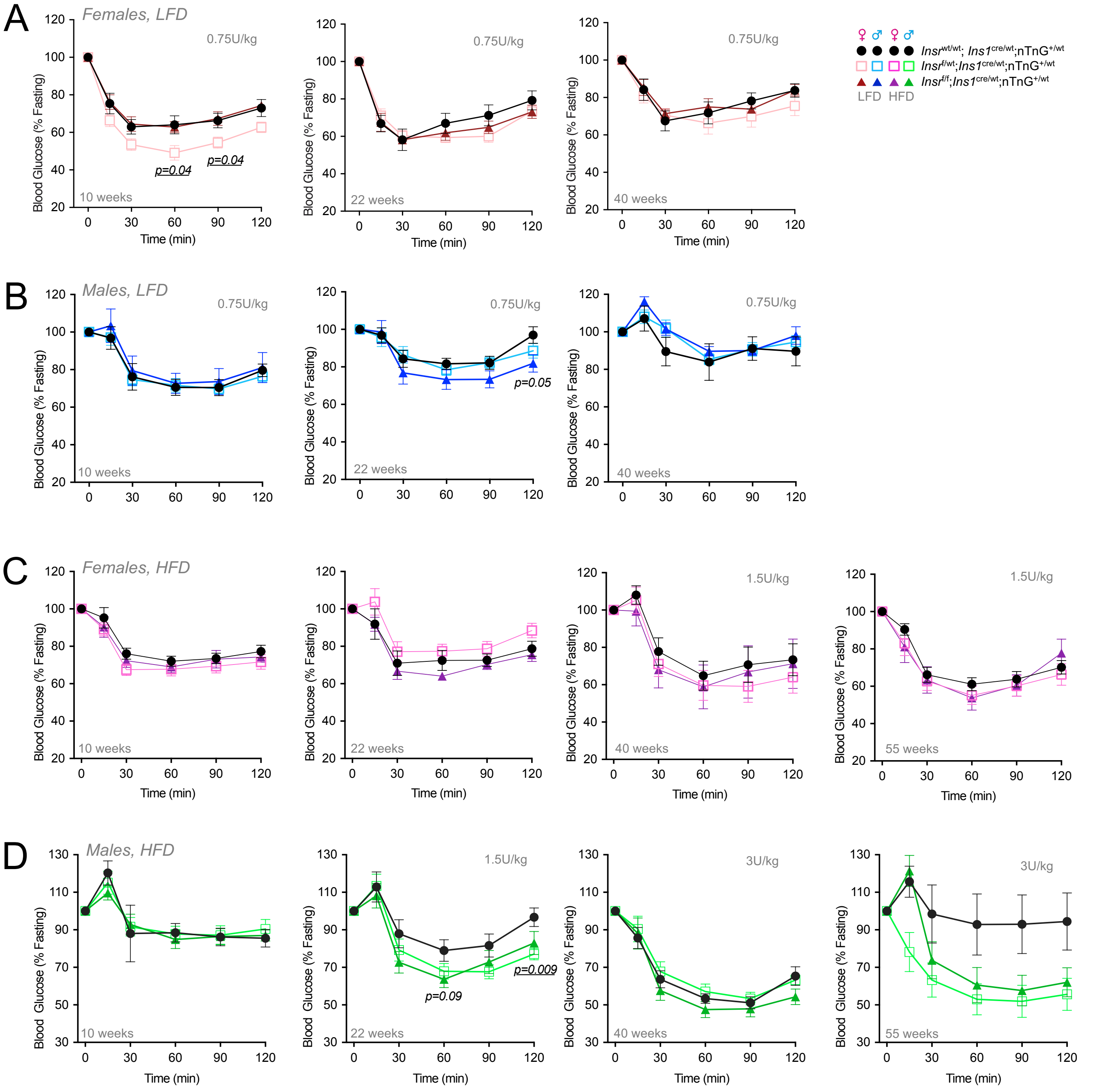

Fig. S6. Insulin tolerance tests. (A-D) Insulin tolerance tests after a 6 hour fast in control, β*Insr*^{HET} and β*Insr*^{KO} mice fed LFD or HFD at multiple ages (n=5-26). Statistical analyses were done with repeated measures 2-way ANOVA. Doses are 0.75 U/kg unless otherwise shown. Data are presented as mean values +/-SEM.

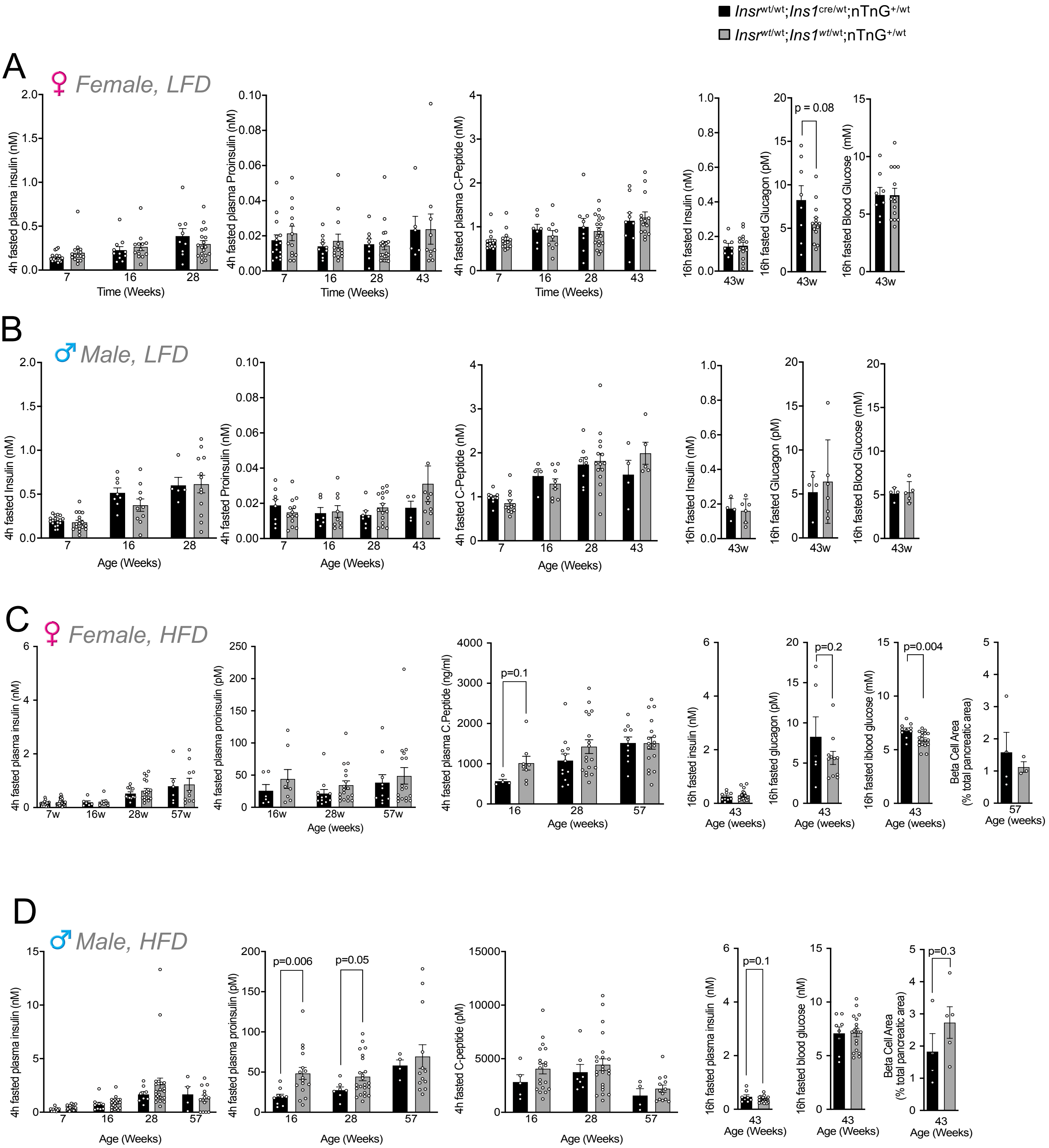

Fig. S7. Fasting insulin, proinsulin, C-peptide and glucagon in control mice. (A-D) Circulating plasma insulin, proinsulin and C-peptide levels after a 4-hour fast, and their associated ratios as well as 16-hour fasted insulin, glucagon and blood glucose levels in *Insr*$^{wt/wt}$;*Ins1*$^{Cre/wt}$;nTnG$^{+/-}$ (*black*) and *Insr*$^{wt/wt}$;*Ins1*$^{wt/wt}$;nTnG$^{+/-}$ (*grey*) LFD-mice at multiple ages (n=3-23). Data were analysed by a fitted mixed-effects model with correction for multiple comparisons using Dunnett's method and 1-WAY ANOVA. Data are presented as mean values +/-SEM.

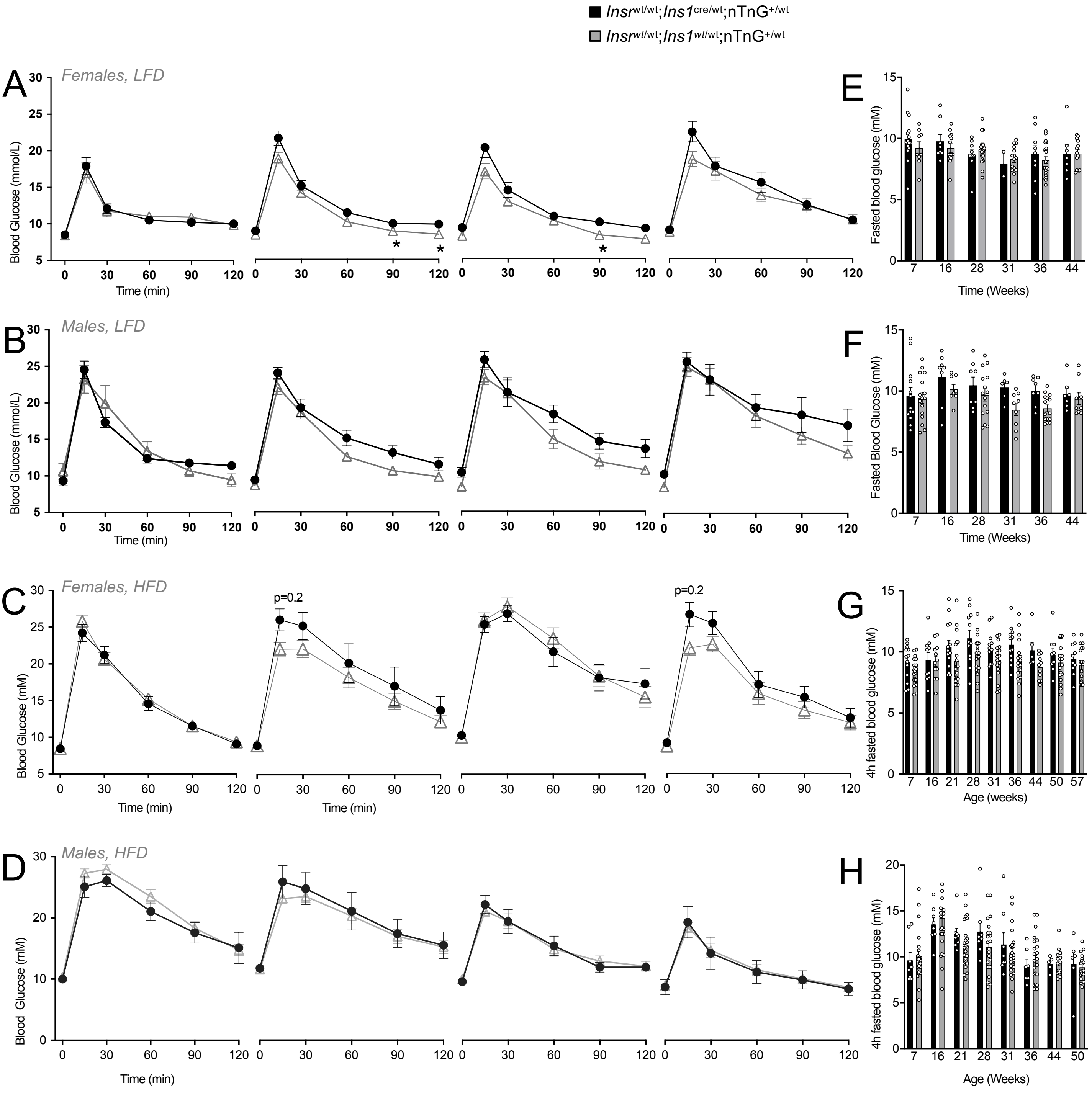

Fig. S8. Glucose tolerance and fasting glucose comparison in controls. (A-D) Glucose tolerance tests after a 6 hour fast in female and male of $Insr^{wt/wt}$;$Ins1^{Cre/wt}$;nTnG$^{+/-}$ (black) and $Insr^{wt/wt}$;$Ins1^{wt/wt}$;nTnG$^{+/-}$ (grey) fed LFD or HFD at multiple ages (n=5-30). Statistical analyses were done with repeated measures 2-way ANOVA. **(E-H)** Blood glucose after a 4 hour fast. Data are presented as mean values +/-SEM.

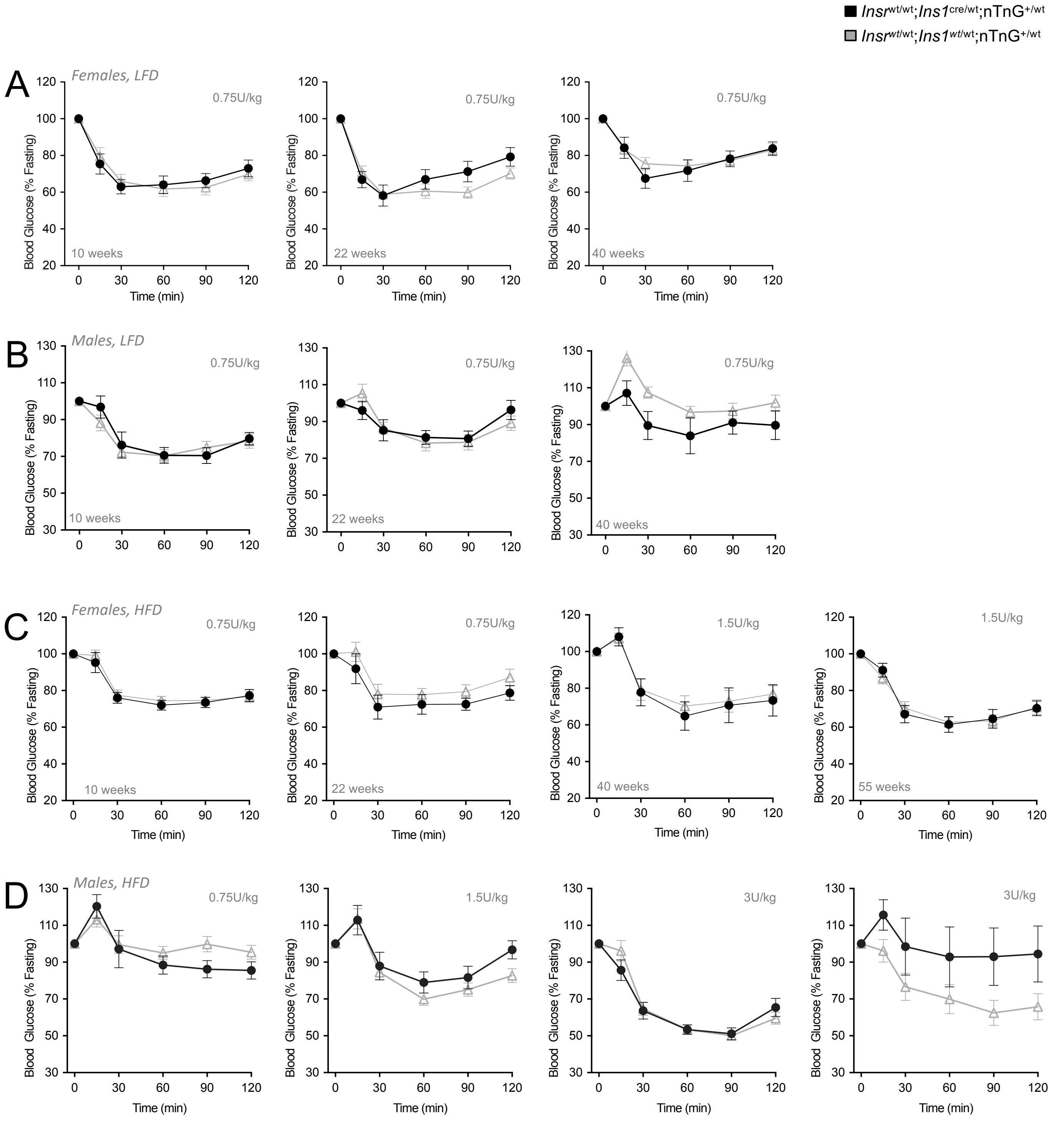

Fig. S9. Insulin tolerance tests in control mice. (A-D) Insulin tolerance after a 6 hour fast in female and male of *Insr*$^{wt/wt}$;*Ins1*$^{Cre/wt}$;nTnG$^{+/-}$ (black) and *Insr*$^{wt/wt}$;*Ins1*$^{wt/wt}$;nTnG$^{+/-}$ (grey) fed LFD or HFD at multiple ages (n=5-30). Statistical analysis were done with repeated measures 2-way ANOVA. Doses are 0.75 U/kg unless otherwise shown. Data are presented as mean values +/-SEM.

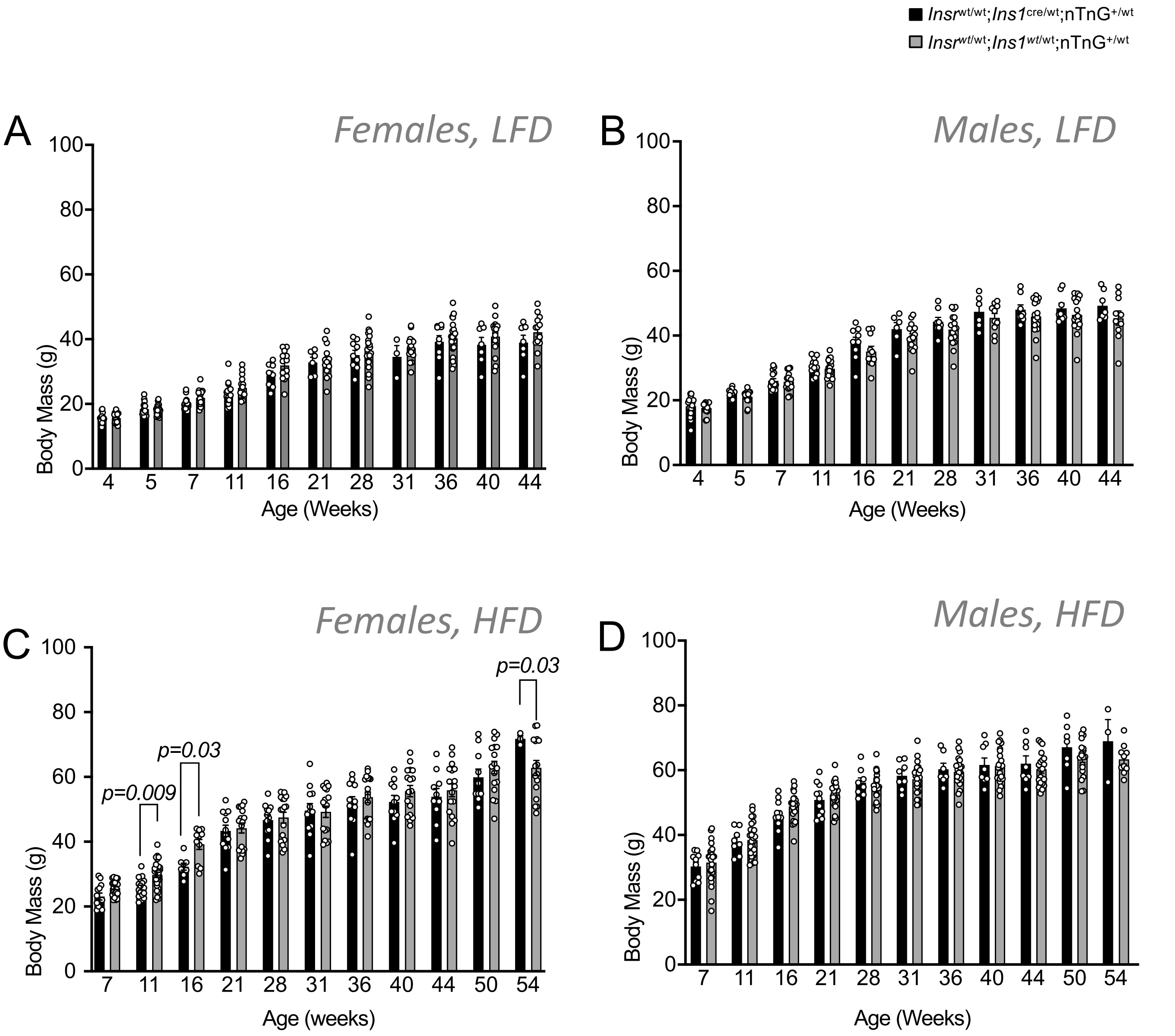

Fig. S10. Body weight in control mice. (A-D) Body weight in female and male of *Insr*$^{wt/wt}$;*Ins1*$^{Cre/wt}$;nTnG$^{+/-}$ (black) and *Insr*$^{wt/wt}$;*Ins1*$^{wt/wt}$;nTnG$^{+/-}$ (grey) fed LFD or HFD at multiple ages (n=4-29). Statistical analysis with mixed effect model. Data are presented as mean values +/-SEM.

Table S1. parameter values for mathematical model

Name	Value (units)	Description (from Topp et al 2000 except *)
R_0	555 mM d^{-1}	Net rate of glucose production at zero blood glucose
E_{G0}	0.5 d^{-1}	Total glucose effectiveness at zero blood insulin
S_P	69.4 d^{-1} nM^{-1}	Total peripheral insulin sensitivity
σ_0	0.864 nM $mg^{-1}d^{-1}$	Maximal rate of insulin release from β-cells
S_β	3.36 nM^{-1}	* β-cell insulin sensitivity to insulin
α	154.0 (12.41) mM^2	Glucose level at half-maximal insulin secretion rate
k	400 d^{-1}	Clearance rate of insulin from the blood
d_0	0.06 d^{-1}	β-cell death rate at zero glucose
r_1	0.0151 $mM^{-1}d^{-1}$	β-cell growth rate due to glucose
r_2	7.792x10^{-4} $mM^{-2}d^{-1}$	β-cell death rate due to glucose

Table S2. List of antibodies.

Primary antibody (immunuflourescence)	Vendor	Catalog number
Glucose Transporter 2	Millipore	07-1402
Insulin	Abcam	ab7842
Insulin	Dako	A0564
anti-glucagon	Cell Signaling Technologies	2760S
anti-BrdU	Abcam	ab6326
**Secondary antibody (immunuflourescence)**	**Vendor**	**Catalog number**
anti-rabbit IgG Alexa Fluor 488	Thermo Fisher Scientific (Invitrogen)	A-11008
anti-Guinea Pig IgG Alexa Fluor 594	Thermo Fisher Scientific (Invitrogen)	A-11076
anti-rabbit Alexa-488	Thermo Fisher Scientific (Invitrogen)	A11034
anti-rat Alexa-594	Thermo Fisher Scientific (Invitrogen)	A11007
anti-guinea pig Alexa-647	Thermo Fisher Scientific (Invitrogen)	A21450
anti-guinea pig Alexa-594	Thermo Fisher Scientific (Invitrogen)	A-11076
**Primary antibodies (Western blot)**	**Vendor**	**Catalog number**
INSR-β subunit	Cell Signaling Technologies	3020S
ERK1/2	Cell Signaling Technologies	#4695
p-ERK1/2	Cell Signaling Technologies	#4370
AKT	Cell Signaling Technologies	#9272
p-AKT	Cell Signaling Technologies	#9275

Antibody validation:
Anti bodies from Cell Signaling Technologies are validated, in house, in multiple research applications (https://www.cellsignal.com/about-us/cst-antibody-performance-guarantee)

Invitrogen antibodies purchased through Thermo Fisher are undergoing a rigorous 2-part testing approach (https://www.thermofisher.com/ca/en/home/life-science/antibodies/invitrogen-antibody-validation.html?icid=ab-search-additional-resources-ab-validation)

Abcam applies an in-house antibody validation incorporates several advanced technologies a central element of our programme includes the routine use of knockout validation that employs CRISPR gene-edited KO cell lines to provide “true” negative controls. This initiative was recognised with a 2020 CiteAb award for KO validation. https://corporate.abcam.com/abcam-triumphs-with-industry-award-for-knockout-antibody-validation-success/

The insulin primary antibody from Dako (agilent) (A0564) is routinely validated https://www.agilent.com/cs/library/msds/SDS345_NAEnglish.pdf

Milipore Sigma are employing validation of their antibodies through technologies such as These efforts and collaborations have led to new validation techniques and novel antibody-based technologies, such as improved bead-based multiplex assays and imaging flow cytometry. https://www.emdmillipore.com/CA/en/life-science-research/antibodies-assays/antibodies-overview/Antibody-Development-and-Validation/cFOb.qB.8McAAAFOb64qQvSS,nav

Table S3. Bayes Factors for comparisons in Figure 8.

Contrast	Age (weeks)	Time (minutes)	Difference in Blood Glucose (mM)	95% Credibility Interval (mM) Lower Bound	Upper Bound	Bayes Factor	Evidence
LFD - Female							
	9	15	−2.4	−4.0	−0.7	112	Decisive
	39	15	−4.7	−7.0	−2.4	2,856	Decisive
	4	15	−1.8	−3.6	0.0	20	Strong
	21	15	−3.1	−5.0	−1.2	263	Decisive
	39	15	−3.9	−6.1	−1.7	596	Decisive
	39	30	−2.3	−4.5	−0.1	22	Strong
	39	60	−2.3	−4.5	−0.1	22	Strong
LFD - Male							
	4	30	−2.9	−5.6	−0.2	25	Strong
	39	90	−4.0	−6.6	−1.4	190	Decisive
	39	120	−5.8	−8.4	−3.2	13,332	Decisive
	39	30	−3.6	−5.9	−1.3	227	Decisive
	39	60	−3.9	−6.1	−1.6	455	Decisive
	39	90	−2.9	−5.1	−0.7	59	Very strong
	39	120	−3.8	−6.0	−1.6	420	Decisive
HFD - Female							
	21	15	−4.7	−7.5	−1.8	245	Decisive
	54	30	−5.0	−8.4	−1.6	134	Decisive
	39	90	3.6	0.8	6.4	59	Very strong
HFD - Male							
	54	15	−3.8	−7.3	−0.3	26	Strong

If no result is reported, the posterior probability did not exceed 95%.
