# Supplementary material for: Beta-cell specific Insr deletion promotes insulin hypersecretion and improves glucose tolerance prior to global insulin resistance
Source: Nat Commun. 2022 Feb 8;13:735. doi: 10.1038/s41467-022-28039-8 (PMC8826929; doi:10.1038/s41467-022-28039-8)
Supplement: Supplementary file 3 — Reporting Summary [file 41467_2022_28039_MOESM3_ESM.pdf]

nature portfolio

Corresponding author(s): James D. Johnson
Last updated by author(s): Nov 19, 2021

# Reporting Summary

Nature Portfolio wishes to improve the reproducibility of the work that we publish. This form provides structure for consistency and transparency in reporting. For further information on Nature Portfolio policies, see our Editorial Policies and the Editorial Policy Checklist.

## Statistics

For all statistical analyses, confirm that the following items are present in the figure legend, table legend, main text, or Methods section.

| n/a | Confirmed | |
|---|---|---|
| ☐ | ☒ | The exact sample size ($n$) for each experimental group/condition, given as a discrete number and unit of measurement |
| ☐ | ☒ | A statement on whether measurements were taken from distinct samples or whether the same sample was measured repeatedly |
| ☐ | ☒ | The statistical test(s) used AND whether they are one- or two-sided<br>*Only common tests should be described solely by name; describe more complex techniques in the Methods section.* |
| ☒ | ☐ | A description of all covariates tested |
| ☐ | ☒ | A description of any assumptions or corrections, such as tests of normality and adjustment for multiple comparisons |
| ☐ | ☒ | A full description of the statistical parameters including central tendency (e.g. means) or other basic estimates (e.g. regression coefficient) AND variation (e.g. standard deviation) or associated estimates of uncertainty (e.g. confidence intervals) |
| ☐ | ☒ | For null hypothesis testing, the test statistic (e.g. $F$, $t$, $r$) with confidence intervals, effect sizes, degrees of freedom and $P$ value noted<br>*Give P values as exact values whenever suitable.* |
| ☐ | ☒ | For Bayesian analysis, information on the choice of priors and Markov chain Monte Carlo settings |
| ☒ | ☐ | For hierarchical and complex designs, identification of the appropriate level for tests and full reporting of outcomes |
| ☒ | ☐ | Estimates of effect sizes (e.g. Cohen's $d$, Pearson's $r$), indicating how they were calculated |

*Our web collection on statistics for biologists contains articles on many of the points above.*

## Software and code

Policy information about availability of computer code

| | |
|---|---|
| Data collection | Detailed description on data collection can be found in the methods section. SlideBook 6 Digital microscopy software (Denver, CO) was used to collect live cell images of cellular calcium levels. |
| Data analysis | Prism 6.0h (GraphPad Software Inc., version 9, San Diego, CA); Adobe Photoshop software version 7; PatchMaster Software version 2X91 (HEKA Instruments Inc, Lambrecht/Pfalz, Germany); Cell profiler automated counting software from Broad Institute (Cambridge, MA); MetaXpress software version 4 (Molecular Devices, San Jose, CA, USA); Fiji 2.1.0-/1.53c image analysis package. R version 4.1.0; R packages: EnhancedVolcano; gplots (heatmap.2 function); DESeq2; ggplot2; brms; gt; panc8.SeuratData; Seurat. Custom made R codes are deposited at github and can be found here: [https://github.com/caraee/BIRKO];[https://github.com/hcen/birko];[https://github.com/caraee/BIRKO_Ca], [https://github.com/krfaulkner/beta-cell-insulin-resistance], and [https://github.com/caraee/BIRKO]. The web application Biorender was used to generate Figure 2e,f and S1. |

For manuscripts utilizing custom algorithms or software that are central to the research but not yet described in published literature, software must be made available to editors and reviewers. We strongly encourage code deposition in a community repository (e.g. GitHub). See the Nature Portfolio guidelines for submitting code & software for further information.

## Data

Policy information about availability of data

All manuscripts must include a data availability statement. This statement should provide the following information, where applicable:

- Accession codes, unique identifiers, or web links for publicly available datasets
- A description of any restrictions on data availability
- For clinical datasets or third party data, please ensure that the statement adheres to our policy

All data necessary to interpret and extend the research in of this study are available within this article, in the supplementary source data file. Fig. 1-10 have associated raw data.
The human islet scRNA, calcium, Bayesian IPGTT data source files generated in this study have been deposited in the github database and are available at [https://github.com/caraee/BIRKO]; [https://github.com/caraee/BIRKO_Ca], and [https://github.com/caraee/BIRKO] respectively.
The Raw RNAseq data generated in this study have been deposited in the GEO database under accession code GSE159527 [https://www.ncbi.nlm.nih.gov/geo/query/acc.cgi?acc=GSE159527].

# Field-specific reporting

Please select the one below that is the best fit for your research. If you are not sure, read the appropriate sections before making your selection.

- [x] Life sciences
- [ ] Behavioural & social sciences
- [ ] Ecological, evolutionary & environmental sciences

For a reference copy of the document with all sections, see nature.com/documents/nr-reporting-summary-flat.pdf

# Life sciences study design

All studies must disclose on these points even when the disclosure is negative.

| | |
|---|---|
| Sample size | Sample size were based on experience with the chosen methods used. We were guided by power calculations in the cases of the large 'primary' endpoints such as glucose tolerance, body weight. |
| Data exclusions | No data exclusions. |
| Replication | We employed multiple orthogonal experiments to ensure the reproducibility of our findings. |
| Randomization | Allocation into groups were based on genotype and the specific animals chosen per group was randomized. |
| Blinding | All studies that involved manual scoring/analysis or hands-on work were conducted in a strictly blinded manner. |

# Reporting for specific materials, systems and methods

We require information from authors about some types of materials, experimental systems and methods used in many studies. Here, indicate whether each material, system or method listed is relevant to your study. If you are not sure if a list item applies to your research, read the appropriate section before selecting a response.

### Materials & experimental systems

| n/a | Involved in the study | |
|---|---|---|
| ☐ | ☒ | Antibodies |
| ☒ | ☐ | Eukaryotic cell lines |
| ☒ | ☐ | Palaeontology and archaeology |
| ☐ | ☒ | Animals and other organisms |
| ☒ | ☐ | Human research participants |
| ☒ | ☐ | Clinical data |
| ☒ | ☐ | Dual use research of concern |

### Methods

| n/a | Involved in the study | |
|---|---|---|
| ☒ | ☐ | ChIP-seq |
| ☒ | ☐ | Flow cytometry |
| ☒ | ☐ | MRI-based neuroimaging |

## Antibodies

| | |
|---|---|
| Antibodies used | See the antibody table S2 and the Methods section for details on antibody dilutions, company names and catalogue numbers. |
| Validation | See antibody table S2 for details:<br>Antibody validation:<br>Anti bodies from Cell Signaling Technologies are validated, in house, in multiple research applications |

(https://www.cellsignal.com/about-us/cst-antibody-performance-guarantee)

Invitrogen antibodies purchased through Thermo Fisher are undergoing a rigorous 2-part testing approach (https://www.thermofisher.com/ca/en/home/life-science/antibodies/invitrogen-antibody-validation.html?icid=ab-search-additional-resources-ab-validation)

Abcam applies an in-house antibody validation incorporates several advanced technologies a central element of our programme includes the routine use of knockout validation that employs CRISPR gene-edited KO cell lines to provide "true" negative controls. This initiative was recognised with a 2020 CiteAb award for KO validation. https://corporate.abcam.com/abcam-triumphs-with-industry-award-for-knockout-antibody-validation-success/

The insulin primary antibody from Dako (agilent) (A0564) is routinely validated https://www.agilent.com/cs/library/msds/SDS345_NAEnglish.pdf

Milipore Sigma are employing validation of their antibodies through technologies such as These efforts and collaborations have led to new validation techniques and novel antibody-based technologies, such as improved bead-based multiplex assays and imaging flow cytometry.
https://www.emdmillipore.com/CA/en/life-science-research/antibodies-assays/antibodies-overview/Antibody-Development-and-Validation/cFOb.qB.8McAAAFOb64qQvSS,nav

## Animals and other organisms

Policy information about studies involving animals; ARRIVE guidelines recommended for reporting animal research

| | |
|---|---|
| Laboratory animals | This study used mice (mus musculus) that were primarily of the C57/bl6-J background and included genetically engineered genes (Ins1Cre knoc in allele, insr floxed allele, nTnG reporter allele). All Jax catalog numbers and details of the applied breeding scheme for this study can be found in the methods section. Details of Age, Sex, Diet and n can be found in main text, figures and figure legends. |
| Wild animals | The Study did not involve any wild animals |
| Field-collected samples | The Study did not involve any field-collected samples |
| Ethics oversight | University of British Columbia Animal Care Committee; Institutional Animal Care and Use Committee of the University of Massachusetts Medical School; University of Michigan Institutional Animal Care and use Committee. |

Note that full information on the approval of the study protocol must also be provided in the manuscript.
